# Predicting Maximum Lake Depth from Surrounding Topography

**DOI:** 10.1371/journal.pone.0025764

**Published:** 2011-09-30

**Authors:** Jeffrey W. Hollister, W. Bryan Milstead, M. Andrea Urrutia

**Affiliations:** 1 Office of Research and Development, Atlantic Ecology Division, United States Environmental Protection Agency, Narragansett, Rhode Island, United States of America; 2 Department of Natural Resources Science, University of Rhode Island, Kingston, Rhode Island, United States of America; University of Bristol, United Kingdom

## Abstract

Information about lake morphometry (e.g., depth, volume, size, etc.) aids understanding of the physical and ecological dynamics of lakes, yet is often not readily available. The data needed to calculate measures of lake morphometry, particularly lake depth, are usually collected on a lake-by-lake basis and are difficult to obtain across broad regions. To span the gap between studies of individual lakes where detailed data exist and regional studies where access to useful data on lake depth is unavailable, we developed a method to predict maximum lake depth from the slope of the topography surrounding a lake. We use the National Elevation Dataset and the National Hydrography Dataset – Plus to estimate the percent slope of surrounding lakes and use this information to predict maximum lake depth. We also use field measured maximum lake depths from the US EPA's National Lakes Assessment to empirically adjust and cross-validate our predictions. We were able to predict maximum depth for ∼28,000 lakes in the Northeastern United States with an average cross-validated RMSE of 5.95 m and 5.09 m and average correlation of 0.82 and 0.69 for Hydrological Unit Code Regions 01 and 02, respectively. The depth predictions and the scripts are openly available as supplements to this manuscript.

## Introduction

The importance of lake morphometry (e.g. lake depth and lake volume) in understanding the ecology of lake systems has long been recognized [1]. Scientists and managers use this information to describe a lake's residence time, build predictive models of nutrients, pollutants, and ecological populations, and to understand lake productivity. For individual lakes that are the focus of research and management, bathymetry surveys are some of the first data collected. From these data, volume is usually calculated using bathymetric contour maps and planimeters. Lake volume can also be estimated with modern GIS methods if maximum depth is known [2]–[4]. Calculating depth and volume of lakes is a simple task provided bathymetry surveys exist; however, gaining access to these data is often difficult as they are frequently only available as unpublished tables or paper maps. This is especially true in regional studies that include a large number of lakes.

**Figure 1:**
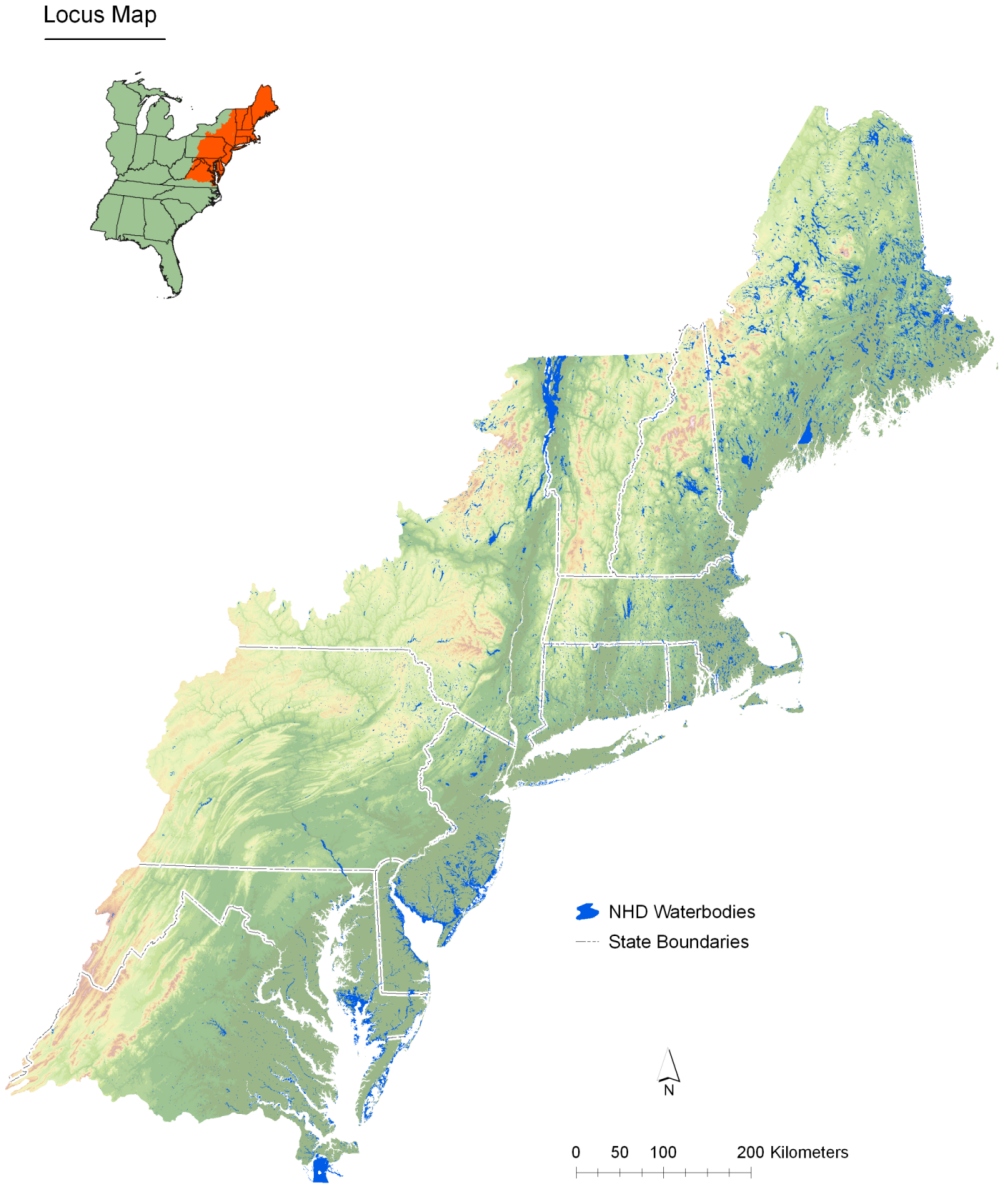
Map of study area showing the Major River Basin 1 Boundary, Hydrologic Unit Code Boundaries, State Boundaries, and Lakes included in this study.

As part of the US Environmental Protection Agency's Ecosystem Services Research Program (ESRP), we are evaluating how changes in nutrient loads impact the delivery of ecosystem services in lakes in the Northeastern Region of the United States (Figure 1) [5]. Obtaining bathymetry data for even a small percentage of the lakes within this region has been difficult and has forced us to model lake depth and volume using existing, publically available datasets. One key source of information that provides insight into lake depth is the National Elevation Dataset (NED) [6]. With this information it is possible to calculate changes in elevation surrounding lakes, which is likely similar to the change in depth within lakes as the same processes formed the surrounding topography and the lake basin [1]. Thus, we assume that lake basins surrounded by steep topography are likely to have a steeper slope and greater changes in depth than do lake basins with lower topographic relief.

There are two goals of our research. First we develop a method to estimate maximum lake depth for all National Hydrography Dataset Plus (NHDPlus) lake polygons in the Northeast U.S. (USGS Major River Basin 1) with sufficient accuracy for regional scale studies of nutrient cycling and ecosystem services. Lastly, we make the predicted depth predictions, as well as the scripts used to generate those predictions freely available.

## Methods

### Study Area and Data

The development of a method to predict lake depth was an essential part of a larger project examining ecosystem services in lakes and ponds of the Northeastern United States. This larger project uses the USGS SPARROW model recently completed for Major River Basin 1 [7], [8]. Thus, our methods were developed with elevation and lake data from Major River Basin 1 (MRB1) which correspond to NHD HUC regions 01 and 02 (Figure 1). We acquired the NED and NHDPlus datasets for these regions. The NED is a nationally consistent, seamless digital elevation model derived from the best available sources (i.e. topographic maps, remotely sensed, etc.). The NHDPlus is 1∶100,000 scale national hydrography data and includes streams lines, lakes, wetlands, and other hydrologic features. Lakes were selected from the NHDPlus. In some cases a single lake was represented by multiple polygons. The lakes were merged and one of the original IDs was used as a new unique ID and was named the WB_ID. The WB_ID provides a unique identifier for each lake in our dataset. Depth predictions were generated using lake shoreline data from the NHDPlus, reach catchments from NHDPlus, and elevation from the NED. For this study we acquired the 10 meter resolution NED (Data available from U.S. Geological Survey at http://ned.usgs.gov) and resampled that data to a 30 meter resolution. Additionally, we used existing sources of field collected lake depth data to test some key assumptions, and correct and assess the accuracy of the depth predictions. Maximum lake depth measurements from the USEPAs National Lake Assessment (NLA) were used to adjust the initial depth predictions and validate the adjusted predictions [4]. We also assessed the predictions of maximum depth by comparison with maximum depth values for Northeastern lakes collected from internet sources. All data used in this study use the North American 1983 datum and are in an Albers Equal Area Conic projection. The assessment data and predicted depth values are available for download (Dataset S2).

### Depth Prediction

The geophysical processes that shape a lake basin are the same as the geophysical processes that shape the topography directly surrounding that basin [1]. Thus, it is reasonable to expect that the slope of the surrounding topography approximates the slope of the lake bottom. We further assumed that the depth of any given point within a lake is partly a function of the distance from shore since points farthest from shore tend to be deeper than near shore areas [2]. If these two assumptions hold true we can combine distance from nearest shore (D) and the median percent slope of surrounding topography (S_median_) to predict lake depth (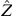) for any pixel within a lake with the following equation:
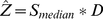
(1)


**Figure 2:**
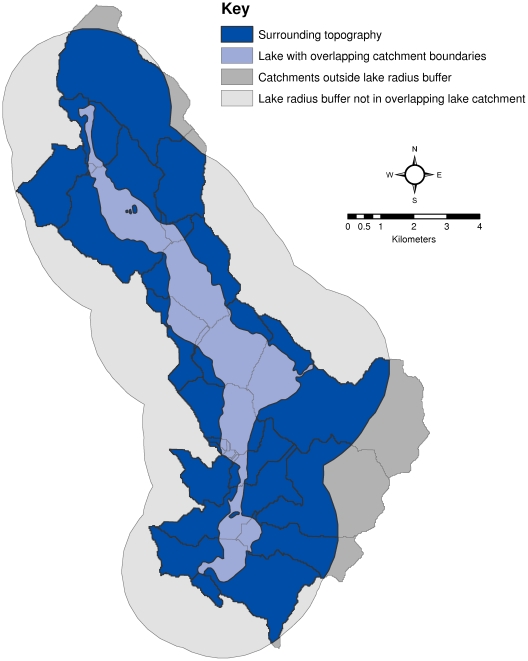
Example map of “surrounding topography” showing lake buffer, overlapping catchments, and the areas that are both within the buffer and overlapping catchment.

Surrounding topography was defined as the land area surrounding a lake that is both within the drainage basin of the lake and is within a specified distance of the lake (Figure 2). The drainage basin was defined to be all NHDPlus catchments that intersect a lake. The specified distance for each lake is the maximum of the in-lake distance to the shore.

To determine 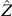
_max_ we used the maximum value calculated with Formula 1. To do this we first calculated the in-lake Euclidean distance from the shoreline for each pixel in the lake and determined the maximum in-lake distance. Second we calculated S_median_ in the surrounding topography using the National Elevation Dataset, re-sampled to 30 meter pixels. Finally, both values were entered into Formula 1 and an initial 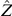
_max_ was recorded for each lake. All analyses were scripted in Python with the geospatial analyses conducted with ArcGIS 10.0, and other statistical and basic database operations conducted with the R programming language [9], [10]. All work can be reproduced with the Python scripts (Text S1) and example data (Dataset S1), assuming the user has access to an ArcGIS 10.0 license and Spatial Analyst. The example data represent a small subset of lakes and may be used to demonstrate the use of the script. The entire dataset is available upon request.

### Adjusting for Bias in Depth Predictions

To determine if a bias existed in our initial 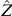
_max_ predictions and, if necessary, to estimate a correction factor, we compared our initial maximum depth predictions from Formula 1 with measured maximum depth for 133 lakes in MRB1 that were sampled as part of the National Lakes Assessment (NLA) in 2007 (Dataset S2). To reduce the impact of influential points in estimating the correction factor, we removed lakes with a Cook's distance greater than 1, a commonly used rule of thumb for identifying influential points [11]. With the remaining 130 lakes, we used a cross-validation technique where 80% of the 130 NLA lakes were randomly selected as a training dataset to compare with our initial predictions and 20% of the lakes were set aside for validation purposes. We used linear regression through the origin on the training dataset to estimate the relationship between our predictions and the NLA measurements. We used the slope of this regression line as a correction factor to empirically correct our initial predictions. The final correction factor was the median of the slope of the regression through the origin for the 10,000 iterations of selecting the 80% training dataset. In each of the 10,000 iterations, we separated HUC regions 01 and 02 and estimated the correction factor independently to account for latitudinal gradients in topography. These methods were used to predict and adjust the maximum lake depth for 27,942 lakes in MRB1.

### Validation of Depth Predictions

To validate the final corrected predictions, we compared the adjusted predicted values to the 20% validation dataset and recorded the RMSE and correlation coefficient. This was done for each of the 10,000 iterations and we report the mean correlation coefficient and the mean RMSE. The validation dataset size of 20% allowed us to maximize the size of the training dataset, while minimizing the variation in both validation metrics. Additionally, we compare the final predictions to an independent source of depth data taken from internet searches. We searched for the names and locations of approximately 600 randomly selected lakes in MRB1. We located maximum depths for 191 lakes (Dastaset S2). We compared our predictions to the reported maximum depths and calculated the RMSE and correlation coefficient.

## Results and Discussion

**Figure 3:**
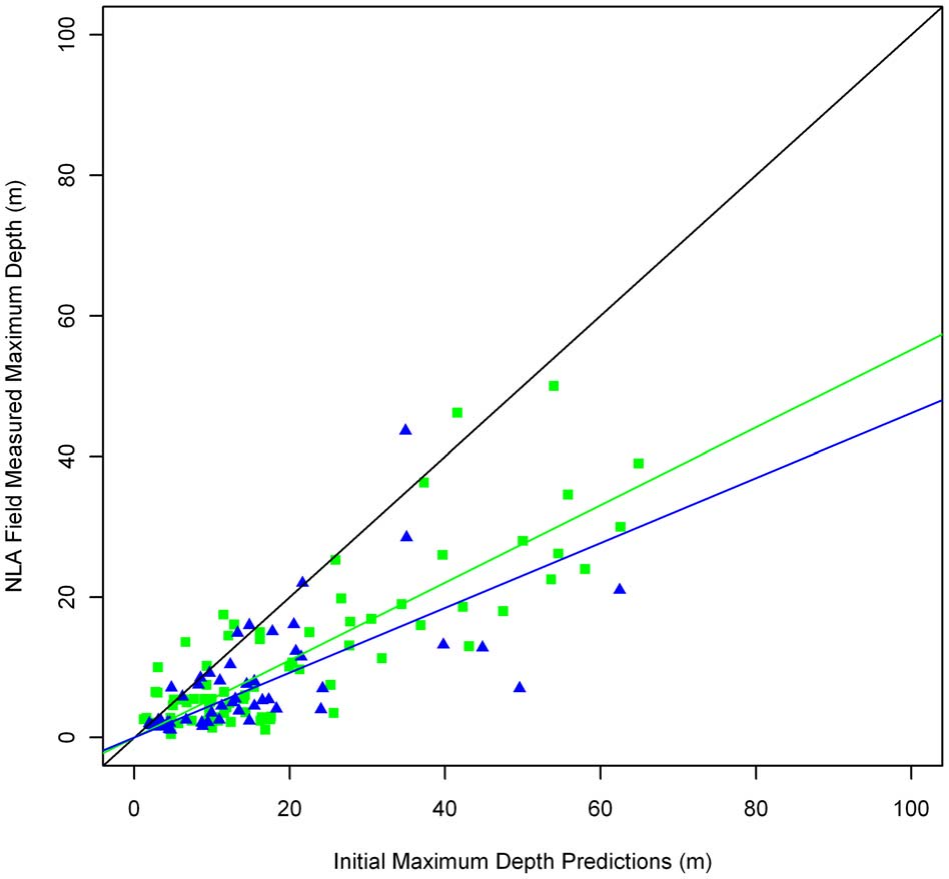
Initial maximum depth predictions compared to National Lakes Assessment (NLA) field measured depths. Black line is one-to-one line indicating perfect agreement. Green squares are values from HUC Region 01 and green line is linear fit with intercept of 0 and slope of 0.553 for HUC Region 01. Blue triangles are values from HUC Region 02 and blue line is linear fit with intercept of 0 and slope of 0.462 for HUC Region 02.

Our initial predictions, when compared to the NLA measured depths, were on average greater than measured values and thus required an adjustment to correct for bias (Figure 3). To correct maximum depths in HUC Region 01 we multiplied initial depth predictions by 0.553 and in HUC Region 02 we multiplied all initial depth predictions by 0.462. These constants are the median slope of the linear fit of the 10,000 cross-validation iterations of the NLA depths and predicted depths with an intercept constrained to 0 (Figure 3). Prior to correcting the estimates, the RMSE for HUC 01 was 6.32 m, and for HUC 02 was 6.14 m. The uncorrected correlation coefficient for HUC 01 was 0.82, and for HUC 02 was 0.64. After corrections, the mean RMSE from the 10,000 validation datasets for HUC 01 was 5.95 m and for HUC 02 it was 5.09 m. The mean correlation for HUC 01 was 0.82 and for HUC 02 it was 0.69. Comparing our predictions to the independent depths from the online sources resulted in an RMSE of 7.0 and a correlation of 0.72. Initial and corrected maximum lake depth predictions for all 27,942 lakes are available for download (Dataset S2). Additionally, the web reported depths and NLA measured depths used for the cross validation are included for the subset of lakes for which those data exist (Dataset S2).

Our primary goal for this research was to develop a method to predict maximum lake depth using publically available data that would be applicable at regional scales. We were successful in that regard and can now estimate lake depth for all lakes with surrounding elevation data, catchments, lake shoreline data (available for the whole United States at the 1∶100 000 scale or better in many cases), and a dataset used to empirically adjust the estimates (i.e. the NLA is available for ∼1000 lakes across the US). Furthermore, our assessment indicates that on average our predictions are accurate, evidenced by the average RMSE and strong average correlation between our predictions and the validation datasets. The methods described here represent a good first step in estimating maximum lake depth when detailed data are not available. There is variation between our estimates and the validation datasets (i.e., cross-validation and web reported depths) that we were not able to explain. There are many possible sources of error that might account for this. We have identified two that we feel to be most important.

First, our uncorrected depth predictions were greater than the measured depths. This suggests processes in addition to those that formed the topography are controlling the depth of the lakes. The most likely processes are erosion and sedimentation. Our method corrects for the over-prediction, but it does not explicitly account for the lake-to-lake variation in sediment loads or age of the lake. Including these loads may account for the over-prediction and should also improve the overall accuracy of the method. One possible addition to this method would be to use existing sediment load models (e.g. USDA's Soil and Water Assessment Tool - SWAT) to estimate sediment loads for each lake [12]–[14].

Second, while the median slope of the surrounding topography does a good job of predicting the average trend in lake depths across a region, the median is likely missing local variations in slope that are important in describing lake bathymetry. Other methods may provide additional information. For instance, interpolation methods (e.g. splines, kriging, polynomial regression) have a long history in generating digital elevation models [15]–[17]. Modifying these techniques so that they may be used to estimate depth in lakes might improve upon a method that uses median slopes.

Lastly, this method makes it possible to greatly expand and enhance modeling studies to include a much larger spatial extent. We can now include volume, via proven GIS methods, and residence time in modeling efforts in a large number of lakes across large regions [2]. In an unpublished study by Milstead and others, they found that including volumes and residence times based on the estimates of depth reported here have improved predictions of nutrient concentrations.

In summary, we developed a method to predict maximum lake depth and lake volume that, on average, had relatively low error (i.e. RMSE ∼5–6 m and strongly correlated with validation data). Unpublished work by Milstead and others also shows that the depth predictions from this method will improve regional modeling studies of lakes and allow for better predictions of nutrient concentration across all lakes in a region. Although our predictions are reasonable, there is room for improvement. Exploring other statistical methods and including estimates of sediment load may provide better estimates. This method is not meant to be a replacement for detailed bathymetric surveys when greater detail is needed. It does allow us to include important information over a much greater number of lakes than was possible previously. Lastly, given wide variation in the age, origin and sedimentary processes seen in lakes of the northeastern United States, that this simple model can explain 50–60% of the variation in maximum depth based solely on a 30 m Digital Elevation Model (DEM) and available *in situ* data is promising and should utlimately improve our ability to model various aspects of lakes across broad regions.

## Supporting Information

Text S1 **Python script to predict maximum lake depths.**
(PY) Click here for additional data file.

Dataset S1 **Example GIS data to be used as input into python script to predict maximum lake depths saved as a self-extracting zip file.**
(EXE)

Dataset S2 **Predicted maximum lake depths for all lakes in Major River Basin 1.** Also includes additional assessment data collected in the field by the National Lakes Assessment, maximum lake depth reported in online sources, and URLs for those sources.
(CSV)
